# Children’s and Adults’ On-Line Processing of Syntactically Ambiguous Sentences during Reading

**DOI:** 10.1371/journal.pone.0054141

**Published:** 2013-01-17

**Authors:** Holly S. S. L. Joseph, Simon P. Liversedge

**Affiliations:** 1 Department of Experimental Psychology, University of Oxford, Oxford, United Kingdom; 2 School of Psychology, University of Southampton, Southampton, United Kingdom; University of Leicester, United Kingdom

## Abstract

While there has been a fair amount of research investigating children’s syntactic processing during spoken language comprehension, and a wealth of research examining adults’ syntactic processing during reading, as yet very little research has focused on syntactic processing during text reading in children. In two experiments, children and adults read sentences containing a temporary syntactic ambiguity while their eye movements were monitored. In Experiment 1, participants read sentences such as, ‘The boy poked the elephant with the long stick/trunk from outside the cage’ in which the attachment of a prepositional phrase was manipulated. In Experiment 2, participants read sentences such as, ‘I think I’ll wear the new skirt I bought tomorrow/yesterday. It’s really nice’ in which the attachment of an adverbial phrase was manipulated. Results showed that adults and children exhibited similar processing preferences, but that children were delayed relative to adults in their detection of initial syntactic misanalysis. It is concluded that children and adults have the same sentence-parsing mechanism in place, but that it operates with a slightly different time course. In addition, the data support the hypothesis that the visual processing system develops at a different rate than the linguistic processing system in children.

## Introduction

Traditionally, research investigating skilled adult syntactic processing during reading [1]–[11] has progressed quite separately from that examining syntax acquisition in children [12]–[14], and indeed most studies which have investigated children’s on-line syntactic processing have been in the domain of spoken language, mainly using the visual world paradigm [15]–[20]. This means that to date, children’s on-line syntactic processing during reading has received little investigation, and as a consequence, while much is understood about the psychological mechanisms and processing preferences that exist for adults, it is not yet known whether similar processes and predispositions underlie children’s moment-to-moment syntactic analysis of written language. The two experiments reported here used eye movement methodology to examine how school-aged children and adults process written sentences which are temporarily syntactically ambiguous. Our central premise is very simple: If children show similar patterns of eye movements and reading time effects as adults in response to particular manipulations of syntactic structure, then it seems likely that the mechanisms they have in place for computing syntactic structure are the same as those found in skilled adult readers. If the nature, time course or magnitude of any such effects is different to that obtained for adults, then this suggests a qualitative and/or temporal difference in processing between the two groups.

When a word is lexically identified during sentence processing, its syntactic category (e.g. noun, verb, adjective, determiner) becomes available, and on the basis of this information, combined with the application of grammatical conventions, the reader computes the structural relations that exist between the constituents of a sentence. How the reader computes the structure of a sentence is known as syntactic processing or parsing, and this process takes place incrementally with the syntactic analysis of the sentence developing as each new word is encountered. Often, sentences, or part-sentences, are syntactically ambiguous; that is there are two or more alternative syntactic interpretations, and much of the evidence for incremental syntactic processing comes from the study of potentially ambiguous sentences [21].

Empirical research has shown that adults exhibit strong preferences in their analysis of certain syntactic ambiguities, under certain conditions, and these biases have been explained over the years by different theoretical accounts: the Garden Path Model [22]; Referential Theory [3]; Constraint Satisfaction accounts [23], [24]; the Unrestricted Race Model [25]; and surprisal accounts [11], [26]. Briefly, the Garden Path model postulates a two-stage parsing process: first, one possible syntactic structure is chosen exclusively according to two structural principles (Minimal Attachment and Late Closure), and only subsequently is this analysis checked against semantic/thematic information. Such an account is modular in that the parser does not make use of non-syntactic information in its assessment of alternative analyses. In contrast, Referential Theory proposes a weakly interactive parsing process whereby multiple syntactic analyses are generated in parallel, and discourse/referential information is used to choose the correct analysis. Constraint-based theories view language comprehension as a parallel, continuous (one-stage) process during which multiple sources of information are used at all times to guide syntactic processing. According to this view, probabilistic constraints are combined rapidly and alternative parallel analyses are weighted on the basis of how compatible they are with these different sources of constraint. A hybrid account, the Unrestricted Race Model [25], [27], [28] proposes simultaneous integration of multiple constraints but selection of syntactic structure is instantaneous rather than dynamic (as in constraint-based models). Finally, surprisal accounts [11], [29] advocate parallel, expectation-based, probabilistic disambiguation in syntactic processing and formalize a linking hypothesis stating that the primary source of difficulty incurred in processing a given word is determined by the degree of update in the preference distribution over interpretations of the sentence that the word requires.

While each of these positions has its advocates, the particular theoretical explanation for such biases is not the issue of primary interest here. Instead, what is important for the purpose of the experiments outlined in this paper is that we know for certain that given appropriately constructed materials, adult readers exhibit a clear and robust syntactic processing preference for a particular type of sentence structure, and that they experience difficulty when a particular word or phrase is encountered. In contrast to this, however, we know very little about the syntactic processing preferences of children during reading. Thus, the use of sentence structures which have been shown to generate robust effects in adult readers, but have been modified to be age appropriate for children, will allow us to observe whether children exhibit disruption for such sentences in a similar way to adults. If this is the case, it seems likely that the processing preferences that are known to exist in adults also exist in children. We will also observe whether the time course of any disruption that occurs in children is delayed in relation to the time course of effects in adults. Finally, we can assess whether children and adults differ in the magnitude of the effects observed.

Several early studies investigating adults’ processing of syntactic ambiguity during reading [1] used sentences with the same syntactic structure as sentences (1a–1b) in Table 1. In these sentences the attachment of a prepositional phrase (*with the long stick*/*with the long trunk*) is temporarily ambiguous such that it can be initially attached high in the syntactic tree so that it modifies the verb phrase (*poked*: VP attachment), or low in the syntactic tree so that it modifies the noun phrase (*the elephant*: NP attachment).

**Table 1:** Experimental stimuli for Experiment 1 (1a–1b) and Experiment 2 (2a–2b).

	REGION 1	REGION 2	REGION 3	REGION 4	REGION 5	REGION 6	REGION 7	REGION 8
1a	The boy	poked	the elephant	with	the long	stick	from	outside the cage.
1b	The boy	poked	the elephant	with	the long	trunk	from	outside the cage.
2a	I think	I’ll wear	the new skirt	I bought	tomorrow.	It’s really	nice.	
2b	I think	I’ll wear	the new skirt	I bought	yesterday.	It’s really	nice.	

For sentences such as (1a–1b), it is well established that adults exhibit longer reading times at the noun of the prepositional phrase in sentence (1b) as compared to the same position in sentence (1a) [1], [2], [22], when such sentences are presented in a neutral context, and when the verb is a so-called action verb (such as *hit*, *poke*, *cut*; see Method section for further details). This effect is often termed the garden path effect. The finding that adults exhibit a garden path effect on the noun in sentence (1a) compared to (1b) shows that adults initially attach the prepositional phrase *with the long stick*/*long trunk* high to the verb *poked* which is the correct analysis in sentence (1a) but not in (1b). Although alternative theoretical accounts of this parsing preference offer different explanations as to why these effects are observed, the different theories are in broad agreement that for such sentences, this processing difference does occur. Thus, this phenomenon exists, is robust, and indicates that (for sentences such as 1b relative to 1a), the adult reader has initially syntactically misanalysed the sentence.

More recently a literature focussing on how children process syntactic ambiguity has emerged. Because (in part) of the difficulty of applying adult experimental procedures to children’s reading, this research has mostly used the visual world paradigm [30], [31] to explore spoken language processing preferences. Trueswell et al. [15] reported a study in which it was found that children showed an overwhelming VP-attachment bias (as opposed to an NP attachment bias) when listening to sentences which were temporarily ambiguous (even if this interpretation was not plausible given the referential context). Also, several other studies [16], [17], [32] have shown that children pursue the interpretation that is consistent with verb bias (i.e. whether a verb more often takes a VP or a NP attachment in natural language, as indexed by a sentence completion task), while adults use both verb bias and referential information. These findings for spoken language interpretation show that in some respects children process sentences in a similar manner to adults, whilst in others they do not. Furthermore, these studies demonstrate that it is possible, in principle, to construct experimental stimuli that induce a VP-attachment processing preference (albeit for spoken sentences) in both adults and children.

The rationale for the second experiment was similar to that for Experiment 1, though we built on our findings from Experiment 1 by testing two different age groups of children. We also used a different syntactic structure, thereby allowing us to evaluate our findings across different syntactic structures. Specifically, in Experiment 2 we examined adults’ and children’s processing of sentences such as (2a–2b) in Table 1. In these sentences, the adverbial phrase (*yesterday*/*tomorrow*) can be attached low to the second verb phrase *I bought* (thereby providing modifying information about when the skirt was purchased) or high to the first verb phrase *I’ll wear* (providing modifying information about when the skirt will be worn).

For sentences like these, adults have been shown to exhibit disruption to processing in sentences such as (2a) as compared to sentences such as (2b) when they are presented in isolation [33]. This effect is caused by adult readers initially attaching the adverb *tomorrow* to the phrase marker associated with the verb phrase *I bought*. The resulting temporal mismatch between the adverb and the verb to which it initially attaches results in the detection of an initial syntactic misanalysis. Again, alternative explanations for this effect have been offered in terms of parsing preferences, multiple constraint-satisfaction, or violations in relation to generated expectations. However, as before, for present purposes, the precise explanation for the effect is not as important as the question of whether the effects are robust and reliable. To summarise, the sentences used in Experiment 2, like those used in Experiment 1, were carefully constructed to induce garden path effects and this was achieved through the attachment of an adverbial phrase high, rather than low, within the syntactic tree. Furthermore, we again investigated whether disruption to processing occurred in two different age groups of children, and how this disruption differed in terms of nature, time course and magnitude, to any disruption observed in adults when the adverb was read.

The main overall aim of the current experiments was to investigate differences in syntactic ambiguity processing in school-age children compared to the performance of adults. Previous research has mostly examined (auditory) processing of such structures in pre-school or very young school-aged children [16], [17], [32], and although one study has examined processing of syntactic ambiguity in children aged 7–9 years [34], it was in the domain of oral language and it is still not clear how the ability to reanalyse a sentence following a garden path develops with age [32]. We chose to examine syntactic processing in children aged 6.5–11 years for several reasons. First, six years was the youngest age we could test participants as we needed them to be able to read sentences relatively fluently. Second, research using offline tasks has shown that that grammatical sensitivity [35] and syntactic awareness [36] both in reading and aural language develop rapidly from the age of six and we wanted to examine developmental progress using a more sensitive on-line measure of processing. Finally, it is less clear to what extent, grammatical processing skills increase after the age 9 [37]. Experiment 2 aimed to examine age-related changes in parsing performance by comparing two age groups of children, those aged 6.5–9 years, and those aged 9.5–11 years, with adults.

In order to address our main question as accurately as possible, we wanted to use a measure of language processing that was naturalistic and very sensitive to processing difficulty. We therefore monitored readers’ eye movements as they read our experimental sentences. Monitoring eye movements during reading provides an extremely sensitive index of on-going comprehension processes [38], [39]. Until recently, it has not been feasible to use eye movement methodology with young children but recent advances in technology have now made this possible [40]. One very clear advantage of this methodology is that children’s eye movements can be monitored while they perform the everyday task of reading. Unlike in methodologies used with younger children, it is unnecessary to introduce an additional task, and therefore reading can progress as normal. In addition, using eye movement methodology with children will allow comparisons not only with adult eye movement data, but also with child data obtained though different methodologies.

While monitoring eye movements during reading in children is a relatively recent development, the existing literature shows that children’s comparatively slow reading can be seen in a number of different eye movement measures: first, children make longer and more frequent fixations, and smaller saccades than adults [41]–[48], indicating that they require longer and more frequent visual samples of text in order to engage in lexical identification processes. In addition, these same studies show that children make more regressive eye movements to re-read previous portions of a text than adult readers, presumably due to increased processing difficulty. Moreover, beginning readers have smaller perceptual and letter identity spans than proficient readers [47], [49], that is, they have fewer words or letters visually available to them during a single fixation (although like adults their perceptual spans extend further to the right than the left in languages such as English). There have been a small number of studies which have manipulated visual or linguistic characteristics of the text being read. The results from these studies have shown that children take longer to process long than short words [41], [44], [45], and words which are low rather than high in frequency [43], [44], [50] and that children exhibit disruption to processing when reading sentences which have anomalous or implausible thematic relations [51]. Interestingly, this last study found that implausibility but not anomaly effects were delayed in children relative to adults, showing that children’s higher levels of linguistic processing operate with a slower time course than adults. However, there have been no studies that have used eye movement methodology to examine syntactic ambiguity effects during reading in children relative to adults.

## Experiment 1

Experiment 1 investigated whether children and adults exhibited similar initial attachment preferences when reading temporarily ambiguous prepositional phrase attachment sentences (see 1a & 1b, Table 1). We predicted that both adults and children would exhibit disruption to processing on the disambiguating noun phrase (*long stick* or *long trunk* in sentences 1a and 1b) when the prepositional phrase attached to the noun phrase (*elephant*) rather than the verb phrase (*poked*). Following previous research showing that the duration of fixations and the frequency of fixations and regressive eye movements, decrease with age [46], [47], we anticipated that overall children would take longer than adults to read sentences making longer fixations shorter saccades and more regressions. We also predicted that there might be a difference in the time course of any garden path effects observed in children, and adults. We base this prediction on the only study which has investigated post-lexical processing in children using eye movements during reading, the Joseph et al. [51] study mentioned above, which found delayed effects of thematic processing in children relative to adults. Specifically, we expected adults to show longer fixation durations on, and more regressions out of, the target region. However, for children we anticipated that such effects might occur later, and be spatially localised to words downstream of the target region, and even observed during second pass measures of reading time. If this were the case, then it would indicate that children were less immediate in their detection of and recovery from a garden path. Following previous eye movement studies which have directly compared adult and child groups [51], it was further anticipated that the magnitude of any disruption effects we might observe would be greater in children than in adults; that is, there would be a developmental decrease in the magnitude of the effects observed.

Finally, we know from previous studies that there is much greater variability in children’s eye movement data than in those of adults and this can obscure effects observed in the adult data when age groups are analysed together [40]. Given our a priori motivation to consider directional patterns of effects for children, it was important to explore effects that occurred in this participant group. Furthermore, we were keen to make comparisons between the adult data from previous research [1] and our adult data to ensure consistency and give us confidence in our “bench mark” analyses against which we could consider the effects for the children. For both the above reasons we decided a priori to analyse the adult data both separately, as well as in conjunction with the child data.

### Method

#### Ethics statement

This research was conducted with the ethical approval of the Ethics Committee at the Department of Psychology, Durham University. Informed oral consent was obtained from each child, in addition to the written consent obtained from parents, after explanation of the procedure of the experiment. Informed written consent was obtained from all adult participants.

#### Participants

Thirty adults and 24 children took part in the experiment. Adults were undergraduate and postgraduate students at Durham University. Children were recruited from local primary schools in the Durham area. The mean age of the child group was 9.0 years (range = 6.5–11.7 years). All children completed the word reading and reading comprehension sections of the Wechsler Objective Reading Dimensions test [52] to assess their reading ability. The mean reading age was 11.5 years (range = 8.6–14.9 years), indicating that the children were precocious readers (note that there was no correlation between reading age and eye movement effects for the children in Experiment 1 or 2). While we intended to analyse the child data as two separate age groups, the relatively small sample size and large variability meant that none of these comparisons were statistically significant. For greater statistical power, we therefore analysed all the child data together in Experiment 1.

#### Materials

Our experimental sentences all contained action verbs which, unlike psychological/perceptual verbs (e.g. *think*, *see*, *feel*) frequently take an instrument, or at least the with-phrases following them are more likely to be attached to the verb phrase [16], [34], [53]. This bias is even more pronounced when the noun is preceded by a definite article [34], as was the case in our experimental stimuli. In addition, sentences were pre-screened carefully with both adults and children to ensure that both groups exhibited the same bias towards a VP interpretation. The pre-screen procedure was carried out with 18 adults and 30 children (aged 7–11years).

The pre-screen procedure was a sentence completion task and was computerised using Macromedia Flash MX software. All participants read written experimental instructions and children received additional verbal instructions. Participants viewed a computer monitor that showed the beginning portion of the proposed experimental sentences (e.g. *The boy poked the elephant with the…*) and were instructed to complete the sentences by typing one or more words. They were instructed that the sentences could be completed in any way they chose but that they had to make sense. Once the participant had typed a response, s/he clicked a button to view the next sentence fragment. There were sixteen sentence fragments in total. All participants understood what was required of them and carried out the task successfully.

In a second procedure, we presented eight adults with all the sentence completions produced in the first pre-screen procedure (166 completions in total) and asked them, “*What or who had the …?”* or *“What or who did the … belong to?”* For example, in the sentence completion, “*The boy poked the elephant with the long stick”,* participants were asked, “*What or who had the stick?”*, and had to circle *the boy* or *the elephant* (or *don’t know* for completions which were either globally ambiguous or indecipherable). On the basis of the second procedure, completions were categorised as high-attached (if more than 70% of completions were rated as high-attached), low-attached (if more than 70% of completions were rated as low-attached) or globally ambiguous/don’t know. Results showed that adults completed the sentences with an instrument (i.e. the completions were high-attached) on 77% of occasions, and children on 92% of occasions. These results show (1) that both groups of participants preferred the VP interpretation of the prepositional phrase in the majority of cases and (2) that this preference was stronger in children than adults, *t*(15) = 2.67, *p*<.05.

We constructed sixteen pairs of sentences, with one high attachment (1a) version the other with a low attachment (1b) version of the sentence (see Table 1). All of the sentence pairs but two were identical apart from the target word (*stick* or *trunk* in sentences 1a and 1b). For the two pairs that were not identical, the adjective preceding the target word differed in each condition (*sharp* vs. *apple*, and *broken* vs. *special*). However, in these two cases, the adjectives were of similar length and frequency, and across all the items there was not a reliable difference between the adjectives for either length or frequency across the two conditions (*t* <1.3, *p*>.2). Sentences were divided into eight regions (see Table 1). Region 1 comprised the subject noun (either a proper name or the definite article and a noun). Region 2 comprised the verb. Region 3 comprised the definite article and a noun phrase. Region 4 was always the word *with*. Region 5 was the pre-target region and comprised the definite article and an adjective. Region 6 was the target region and comprised the noun. Region 7 consisted of one long (four letters or more), or two short, words following the target region. Finally, Region 8 encompassed the remainder of the sentence.

#### Apparatus

Participants’ eye movements were recorded using a head-mounted Eyelink II eye tracker manufactured by SR Research (Mississauga, Canada), as they read sentences from a computer monitor at a viewing distance of approximately 100 cm. The eye tracker was an infrared video-based tracking system with two cameras mounted on a headband that were placed approximately 5 cm from the eyes. Head position was detected by four LEDs attached to the computer monitor, and any head movements were compensated for in the eye movement records. Participants’ eye movements were monitored at a rate of 500 Hz to produce a sequence of fixations with start and finish times. Although participants read binocularly, only the movements of the right eye were monitored.

#### Procedure

Participants sat in a customised chair in front of a computer monitor. The eye tracker was placed on the participant’s head and secured by adjusting two headbands. Two cameras were placed in front of the eyes. Participants undertook a calibration procedure during which they looked at each of three horizontal fixation points. Participants then looked at a fixation box on the left of the screen and the sentence appeared contingent on their gaze. Participants were required to read the sentences normally and then press a button when they had finished reading. The button press terminated the display. If the participant did not press the button within 15 seconds of the sentence appearing, the display was automatically terminated. In addition to the 16 experimental items, 67 (43 for children) filler items (including 16 sentences from Experiment 2, see later), and two practice items at the beginning of the experiment were also presented. Participants were asked to respond to yes/no comprehension questions after 25% of the sentences by pressing a button. The questions were included to encourage participants to read carefully; however, an accurate response did not rely on correct resolution of the syntactic ambiguity. The experimental session lasted approximately 35 minutes in total.

## Results and Discussion

Fixations longer than 1200 ms and shorter than 80 ms were systematically excluded from the data set. Trials in which there was tracker loss or excessive blinking were also excluded. In addition, any trials in which the participant did not fixate either the target region (*stick*/*trunk* in sentences 1a and 1b) or the post-target region (*from* in sentences 1a and 1b) were deleted. Outliers (3 Standard Deviations above or below the mean per subject) were also excluded. Together these exclusions resulted in the elimination of 16.7% of the data. All participants performed very well on the comprehension questions with children answering 93% of questions correctly, and adults answering 97% of questions correctly. All participants answered a minimum of 75% of questions correctly.

The following eye movement measures were calculated for the target and the post-target regions: first fixation duration (the duration of the first fixation made in a region); gaze duration (the sum of all fixations in a region until a saccade out of the region); regression probability (the probability of making a leftward eye movement out of a region before leaving that region to the right); go past time (the sum of all temporally contiguous fixations in a region, including regressive eye- movements to the left of the region, until the point of fixation progresses to the region to the right); and total reading time (the sum of all fixations in a region). Mean reading times and regression probabilities for the target and post-target regions are shown in Table 2. Regressions in (the probability of making a leftward eye movement into a region having already left that region to the right) and second pass reading time (total fixation durations in a region after having left that region to the right) in Regions 2 and 3 are shown in Table 3.

**Table 2:** Mean reading times and regression probabilities for adults and children in the target and post-target regions in high-attached (HA) and low-attached (LA) conditions.

			First fixation duration	Gaze duration	First pass regression out	Go past time	Total time
Target region	Adults	HA	239 (77)	260 (95)	0.07 (0.12)	283 (123)	321 (158)
		LA	232 (66)	262 (92)	0.10 (0.11)	310 (171)	343 (181)
	Children	HA	309 (166)	363 (199)	0.11 (0.13)	465 (389)	513 (404)
		LA	295 (125)	377 (204)	0.10 (0.13)	430 (263)	525 (358)
Post-target region	Adults	HA	269 (137)	339 (201)	0.13 (0.16)	444 (432)	393 (238)
		LA	254 (106)	314 (176)	0.17 (0.14)	500 (439)	451 (295)
	Children	HA	327 (185)	429 (256)	0.14 (0.15)	531 (433)	553 (424)
		LA	307 (149)	444 (261)	0.25 (0.18)	774 (680)	643 (438)

Standard deviations in parentheses.

**Table 3:** Mean reading times and regression probabilities for adults and children for Regions 2 and 3, in high-attached and low-attached conditions.

Region	Measure	Adults		Children	
		High-attached	Low-attached	High-attached	Low-attached
2	Regressions into region	0.15 (0.16)	0.16 (0.19)	0.17 (0.11)	0.19 (0.13)
	2nd pass reading time	261 (179)	295 (193)	468 (314)	373 (203)
3	Regressions into region	0.11 (0.11)	0.14 (0.15)	0.16 (0.12)	0.15 (0.12)
	2nd pass reading time	256 (142)	338 (176)	604 (570)	503 (462)

Standard deviations in parentheses.

### Adult Data

We first analysed the adult data alone in order to examine whether we could replicate effects observed in previous studies with our stimuli which were designed for young children. In the target region, paired t-tests showed no difference in first fixations or gaze durations between high and low-attached conditions (*t*s <1; *p*s >.3). There was no difference in the number of regressions made out of the target region, *t*1(29) = .85, *p* = .40; *t*2(15) = 1.59, *p* = .13. However, adults did show marginally longer go past times in the low than high-attached condition, *t*1(29) = 1.84, *p* = .077; *t*2(15) = 2.31, *p*<.05, *d* = 0.16. To investigate this effect in detail, we divided go past times into time spent reading the target region itself, and time spent reading previous regions. These analyses revealed longer reading times in the low than high-attached condition for the preceding regions, *t*1(7) = 2.49, *p*<.05; *t*2(10) = 2.96, *p* = .01, *d* = 0.61, but no difference on the target region itself (*t*s <1.6, *p*s >.13). This indicates that in the low-attached condition, adults detected a misanalysis on encountering the target region, and then spent more time re-reading the earlier part of the sentence before reading the remainder of the sentence when they had misparsed it than when they had not. Finally, there was no difference in total reading times between conditions (*t*s <1.5; *p*s >.15) in the target region.

In the post-target region, there were no differences in first fixations, gaze durations, go past times or regressions made out of the region (*t*s <1.75; *p*s >.09), nor in total reading times, *t*1(29) = 1.58, *p* = .14; *t*2(15) = 2.82, *p*<.05. Given the go past re-reading effects that we obtained in the target region, we were also interested in whether adults made more regressions into, or spent longer re-reading regions preceding the critical word (Regions 2, *poked* in Table 1, or 3 *the elephant* in Table 1) which were the two possible attachment sites for the prepositional phrase. Consistent with the go past reading time data, these analyses showed that adults spent marginally longer re-reading Region 3 in the low than high-attached condition, *t*1(12) = 2.72, *p*<.05; *t*2(12) = 1.90, *p* = .08, *d = *0.58. There were no other reliable differences (*t*s <1; *p*s >.4).

In summary, the analyses of the adult data alone show that they exhibited disruption to processing in the low relative to the high-attached condition. Specifically, having encountered the disambiguating target word, adults spent longer re-reading previous portions of text (in particular Region 3) before continuing to read the remainder of the sentence when a prepositional phrase was attached low to a noun phrase rather than high to a verb. In particular, adults re-read the noun phrase that was the correct site for prepositional phrase attachment, presumably reflecting recovery from their initial misanalysis, or perhaps reanalysis processes. Note that these effects are comparatively small, indicating that the disruption that the adults experienced was minimal.

Our results differ somewhat from those of the Rayner et al. study [1] as they found reliable differences between high and low-attached conditions in gaze duration and total time on the target word (although their reading time analyses were calculated per character). Also, as was the convention at the time of publication, Rayner et al. did not report go past times or regressions. The effects observed in our data are therefore slightly delayed relative to those of Rayner et al., occurring significantly in go past (re-reading) times rather than gaze duration, and not lasting as long, given that the effects were not apparent in the later measure of total time. It is very likely that these differences are due to the relative ease of our experimental sentences which were designed for our child participants (for further discussion of this issue see [40]). Nevertheless, like Rayner et al., we did find clear effects of attachment in the predicted direction.

### Children and Adults

We then analysed the adult and child data together. We conducted a 2 (group: adults, children)×2 (attachment: high-attached, low-attached) mixed design ANOVA. In the target region, there was an effect of group in every reading time measure, with children showing longer first fixations, gaze durations, go past times, and total word reading times than adults (*F*s >18; *p*s <.05), but there was no difference between groups in the number of first pass regressions made out of the target region (*F*s <1; *p*s >.3). These effects are very robust and entirely consistent with existing studies investigating children’s reading [40]. As reported in these studies, children make more and longer fixations, spend more time re-reading sentences and spend longer in total reading sentences.

For the main effect of attachment, there was a trend towards longer first fixations in the high than low-attached condition, but this was not reliable, *F*1(1, 52) –3.38, *p* = .07; *F*2(1, 15) = .41, *p* = .5. There was no interaction in this measure (*F*s <1, *p*s >.3). There were no effects of attachment in gaze durations, regressions out, go past times or total reading times in this region (*F*s <1, *p*s >.6). However, there was an interaction between group and attachment in go past times that was reliable by participants but not by items, *F*1 (1, 52) = 5.36, *p*<.05; *F*2 (1, 15) = 1.97, *p* = .18. No other reliable interactions occurred in this region: *F*s <1.8, *p*s >.2. As reported in the previous section, adults showed marginally longer go past times in the low than the high-attached condition, while children showed no reliable difference between high and low attached conditions, (ts <1.5, ps >.14), showing increased immediacy of ambiguity detection in adults relative to children.

We then analysed the data for the post-target region (see Table 2). Again, consistent with previous studies, there were strong effects of group in this region for all reading time measures with longer reading times for children than for adults (all *F*s >7; *p*s <.01). As in the target region, there was no difference between groups in the number of regressions made (*F*s <2.3, *p*s >.14). There were no effects of attachment and no interactions in first fixation durations or gaze durations (*F*s <1.8, *p*s >.18). However, there was an effect of attachment on the number of first pass regressions made out of the post-target region reliable by participants and very close to significance by items *F*1(1, 52) = 7.22, *p*<.05, η^2^ = .12; *F*2(1, 15) = 4.10, *p* = .06, η^2^ = .21. Readers made more regressions in the low than high-attached condition. There was no interaction in this measure (*F*s <1, *p*s >.3).

There was also a highly reliable and complementary main effect of attachment in go past times, *F*1 (1, 52) = 8.10, *p*<.01, η^2^ = .14; *F*2(1, 15) = 9.37, p<.01, η^2^ = .38, with longer reading times in the low than high-attached condition. There was also an interaction in this measure, *F*1(1, 52) = 3.90, *p* = .05, η^2^ = .07; *F*2 (1, 15) = 5.57, *p*<.05, η^2^ = .27. Pairwise comparisons showed that for children, go past times were significantly longer in the low than high-attached condition, *t*1(23) = 2.47, *p*<.05; *t*2(15) = 2.88, *p*<.05, d = 0.37, however, for adults they were not. Thus, children spent longer than adults processing the post-target word and previous portions of the sentence in the low-attached relative to the high-attached condition. Finally, there was a reliable main effect of attachment in total reading time in the post-target region, *F*1(1, 52) = 7.00, *p*<.05, η^2^ = .12; *F*2(1, 15) = 4.73, *p*<.05, η^2^ = .24, with longer total reading times in the low than high-attached condition, but no interaction (*F*s <1; *p*s >.4). These measures indicate that both children and adults experienced more difficulty processing low than high attached sentences, initially at the post-target word, reflected in increased regressive eye movements. They also indicate that children spent longer re-reading previous portions of sentences before moving on after the initial disruption than did adults, suggesting that their recovery from initial misanalysis was slower than that for adults.

Once again, to scrutinise re-reading behaviour in more detail, we examined times for Regions 2 (*poked*) and 3(*the elephant*). We examined two measures: regressions made into the region, and second pass reading times. In both Regions 2 and 3, we found no effect of group in regressions into the region (*F*s <1.2; *p*s >.3), but a reliable effect of group in second pass reading times (*p*s <.05). In Region 2, we found no effect of attachment and no interaction in the number of regressions made into the region (*F*s <1, *p*s >.7), and no effect of attachment (Fs <2.3, ps >.15) and no reliable interaction in second pass reading times, *F*1(1, 30) = 1.83, *p* = .19; *F*2(1, 12) = 3.89, *p* = .07. Likewise in Region 3, we found no effect of attachment and no interaction in the number of regressions made into the region (*F*s <1, *p*s >.4), no main effect of attachment in Region 3 (*F*s <1, *p*s >.4), and no interaction in second pass reading times, *F*1(28) = 2.73, *p* = .11, η^2^ = .06; *F*2(9) = .96, *p* = .35, η^2^ = .25. We therefore found no evidence of increased regressions into, or reading times in, the two possible attachment sites of the verb. Although the adult analyses did show marginally longer re-reading times in Region 3 then, the overall analyses did not.

In summary, it was predicted that both participant groups would exhibit a preference for attaching the prepositional phrase high to the verb, rather than low to the noun phrase, and that children would show delayed effects of attachment that were of increased magnitude as compared to adults. Consistent with these predictions, adults spent longer re-reading previous portions of the sentence after fixating the target word and before going past it in the low than high-attached condition. These data suggest that adults made a syntactic commitment to attach the prepositional phrase to the verb quite immediately. The same was not true for the children as there was no comparable main effect at this region. However, reduced reading times for high than low-attached sentences did occur for the children in the next region, the post-target region, suggesting that commitment to a high-attached analysis was delayed relative to the adults. There is some suggestion that the effects observed in children were of a greater magnitude than those seen in adults: children made relatively more regressions (11%; cohen’s *d* = 0.47) out of the post-target word in the low than high-attached condition as compared to adults (4%; *d* = 0.21). Furthermore, the data suggest that adults and children may differ in how they attempted to resolve their initial misanalysis. While adults spent marginally longer re-reading the noun phrase to which the prepositional phrase should have been attached in the low-attachment condition, children did not. It may be then that adults are more efficient than children at recovering from or correcting an initial misanalysis.

These findings are important in three respects. First, they indicate that the nature of processing preferences that exist in children are qualitatively similar to those that exist in adults. That is to say, for the type of sentences that were used in this experiment, it appears that both children and adults prefer to initially attach the prepositional phrase to the main verb high in the syntactic tree. The results also indicate, however, that while the nature of processing preferences is similar in adults and children, the speed with which initial syntactic commitments are made is slower in children than in adults. Furthermore, children appear to be less skilled at directing their attention back to the part of the sentence that is most informative in terms of aiding reanalysis.

One question which Experiment 1 does not address is whether children’s efficiency of syntactic analysis and recovery from misanalysis becomes more adult-like with age. As noted, children’s ages in Experiment 1 ranged from 6.5 to 11 years and clearly reading ability improves substantially between these ages. It may be then that younger, but less-so older children, exhibit delayed effects in the detection of syntactic ambiguities, as well as differential patterns of post-detection repair strategies. To investigate this possibility, and in order to see if the effects from Experiment 1 generalised to a different syntactic structure, we conducted a second experiment in which we divided children into younger and older age groups to examine whether age-related changes in the detection and resolution of syntactic ambiguities could be observed.

## Experiment 2

Experiment 2 investigated children and adults’ syntactic processing of sentences with a different structure. Sentences were syntactically ambiguous such that an adverb could initially be attached either to the clause currently being processed, or instead, to a noun phrase earlier in the sentence (e.g., 2a and 2b, see Table 1). To briefly recapitulate, when sentences like (2a & 2b) are presented to adult readers in isolation (known as a null context [54]), they exhibit a parsing bias to initially attach the adverb (*tomorrow*/*yesterday*) to the phrase currently being processed (*I bought*). This preference results in the reader initially adopting the appropriate syntactic analysis for the late-closure version of the sentence (2b). However, for the early-closure version of the sentence (2a), the parsing preference results in a garden path effect due to the temporal mismatch between the adverb and the preceding verb phrase [33]. Thus, we predicted that in the adult participants we would observe inflated reading times on the disambiguating target word in the early-closure condition (*tomorrow* in Example 2) as compared to the late-closure condition (*yesterday* in Example 2). In line with previous research [33], we also predicted that adults would make more regressions from the disambiguating word to refixate earlier portions of the sentence (e.g., *I bought*) to which they had (wrongly) attached the adverb. In addition, and as in Experiment 1, we took the opportunity to make comparisons between the effects reported by Altmann et al. [54] and the effects obtained from analyses of the present adult data to establish that our experiment replicated their findings, and in order to ensure that our adult data were adequate as a bench mark against which to compare the effects obtained for the children. For this reason, as in Experiment 1, we analysed the adult data separately as well as in conjunction with the child data. In addition, while we predicted that adults would show effects on the target word (*tomorrow*/*yesterday*), we anticipated that children might exhibit effects that were both delayed and greater in magnitude than those we obtained for the adults (based on our findings from Experiment 1), and that similar differences may also exist between the younger children as compared to the older children.

An important aim of Experiment 2 was to examine whether syntactic processing efficiency changes with age. It may be that while younger children are delayed in detecting and resolving a syntactic misanalysis, older children are more adult-like in their on-line processing of garden path sentences. As outlined in the Introduction, in this experiment, we divided the children into two age groups. Previous work [35]–[37] has shown that sensitivity to and awareness of grammar increases until age 9 at which point development appears to slow. By separating children into two age groups (6.5–9 and 9.5–11years), it was possible to investigate whether on-line measures of syntactic processing are in line with these findings.

### Method

#### Ethics statement

See Experiment 1.

#### Participants

Thirty adults and 28 (the same 24 from Experiment 1 plus an additional four children) children took part in Experiment 2. In order to examine effects of age, we used a median split to divide the children into two groups (n = 14 in each group). The younger age group had a mean age of 7.9 years (range = 6.5–9.0 years) and the older group had a mean age of 10.4 (range = 9.5–11.7 years). The mean reading age in the younger group was 10.4 years (range 6.8–14.2 years) and the mean reading age in the older group was 13.3 years (range = 8.6–17.0 years).

#### Materials

Experimental sentences were constructed in which the attachment of an adverbial phrase was manipulated (see 2a and 2b in Table 1). The adverbial phrase was either attached high to the verb phrase *I’ll wear* (*tomorrow*), or low to the verb phrase *I bought* (*yesterday*). Experimental sentences were split in to seven regions. We defined the target region as Region 5 of the sentence (see Table 1), and Region 6 as the spillover region (although note that Region 6 comprised the first one or two words of a separate sentence). Regions 2 and 4 comprised the two verb phrases to which the adverbial phrase could be attached to and so these were also of interest.

#### Apparatus and Procedure

The apparatus and procedure were identical to Experiment 1.

## Results and Discussion

Fixations and trials were excluded according to the same criteria as in Experiment 1. In addition one trial was excluded completely from the analyses due to a typographical error resulting in 15 items for the analyses. In total, 11.3% of the data were excluded. All participants performed well on the comprehension questions with children answering 98.9% of questions correctly, and adults answering 99.5% of questions correctly. All participants answered a minimum of 75% of questions correctly.

First fixation durations, gaze durations, the probability of making a first pass regression out of a region, go past times, total reading times, and second pass reading times were calculated in the target region (Region 5) and the post-target region (Region 6; see Table 4). The probability of making a regression into a region and second pass reading time were also calculated for Regions 2 and 4 (see Table 5). As in Experiment 1, we first analysed the data from the adults to ensure that our stimuli were effective in producing the effects reported in previous studies.

**Table 4:** Mean reading times and regression probabilities for adults, older children and younger children for the target region for early closure and late closure conditions.

		Adults		Older children		Younger children	
		Early closure	Late closure	Early closure	Late closure	Early closure	Late closure
Target region	First fixation durations	239 (84)	224 (66)	272 (105)	294 (150)	296 (146)	294 (133)
	Gaze durations	382 (232)	334 (187)	552 (361)	534 (355)	538 (388)	586 (399)
	First pass regressions out	0.22 (0.22)	0.14 (0.17)	0.27 (0.19)	0.16 (0.15)	0.33 (0.26)	0.18 (0.14)
	Go past times	534 (409)	408 (254)	799 (602)	641 (368)	1004 (875)	872 (880)
	2nd pass reading times	357 (235)	225 (97)	676 (682)	643 (608)	739 (565)	620 (482)
	Total reading times	520 (340)	406 (218)	933 (616)	763 (490)	961 (686)	846 (733)
Post- target region	First fixation durations	248 (95)	250 (100)	271 (144)	261 (123)	301 (151)	295 (133)
	Gaze durations	320 (183)	315 (158)	458 (337)	412 (249)	532 (364)	457 (323)
	First pass regressions out	0.05 (0.08)	0.03 (0.06)	0.17 (0.17)	0.06 (0.13)	0.20 (0.15)	0.11 (0.11)
	Go past times	335 (195)	334 (186)	591 (519)	528 (625)	778 (655)	561 (435)
	2nd pass reading times	339 (251)	282 (173)	418 (300)	442 (450)	408 (313)	369 (221)
	Total reading times	438 (287)	419 (210)	695 (420)	619 (433)	794 (474)	624 (401)

Standard deviations in parentheses.

**Table 5:** Mean reading times and regression probabilities for adults, older children and younger children for Regions 2 and 4, for early closure and late closure conditions.

Region	Measure	Adults		Older children		Younger children	
		Early closure	Late closure	Early closure	Late closure	Early closure	Late closure
2	Regressions into region	0.20 (0.21)	0.20 (0.18)	0.36 (0.24)	0.33 (0.30)	0.30 (0.22)	0.29 (0.20)
	2nd pass reading time	351 (175)	307 (203)	533 (335)	521 (366)	582 (357)	610 (610)
4	Regressions into region	0.30 (0.22)	0.22 (0.21)	0.39 (0.22)	0.30 (0.17)	0.40 (0.23)	0.25 (0.20)
	2nd pass reading time	374 (281)	280 (157)	643 (523)	525 (423)	638 (439)	578 (686)

Standard deviations in parentheses.

### Adults

In the target region, we found significantly longer first fixations, *t*1(29) = 2.76, *p*<.05; *t*2(14) = 2.64, *p*<.05, *d* = 0.23, and gaze durations, *t*1(29) = 2.78, *p*<.01; *t*2(14) = 4.08, *p*<.005, *d* = 0.28, in the early-closure than late-closure condition. This difference demonstrates that, consistent with our predictions, and with the findings of Altmann et al. [33], adults exhibited immediate garden path effects at the disambiguating word for the early-closure sentences compared with the late-closure sentences. Adults also made reliably more regressions out of the target region, *t*1(29) = 2.15, p<.05; *t*2(14) = 2.17, *p*<.05, *d* = 0.44, and exhibited longer go past times for the target region, *t*1(29) = 3.25, *p*<.005; *t*2(14) = 3.16, *p*<.01, *d* = 0.41, in the early-closure than late-closure condition. Further analyses showed that these inflated go past times reflected longer reading times on the target word itself, *t*1(29) = 3.79, *p* = .001; *t*2(14) = 3.64, *p*<.005, *d* = 0.47, rather than longer re-reading times in previous regions before moving on to read the remainder of the sentence (ts <1; ps >.3). Finally, adults showed longer second pass reading times, *t*1(16) = 2.66, *p*<.05; *F*2(14) = 3.53, *p*<.005, *d* = 0.84, and longer total reading times, *t*1(29) = 4.10, *p*<.001; *t*2(14) = 4.86, *p*<.001, *d* = 0.54, in the early-closure than late-closure condition.

Strikingly, there were no reliable differences between the early-closure and late-closure condition for the adults in the post-target region at all (all ts <1; all ps >.3), showing that the effect of closure did not spill over (as in Experiment 1). It appears that recovery from the initial misanalysis occurred before readers moved on to inspect new information to the right of the disambiguating word.

There were two further regions of interest in the experimental sentences: Region 2 (*I’ll wear* in sentences 2a and 2b) and Region 4 (*I bought* in sentences 2a and 2b). These regions were of interest because they were the potential attachment sites of the adverb, and contained the information necessary to compute a temporal match between the tense of each of the verbs and the temporal information associated with the adverb. Thus, if readers need to re-inspect earlier portions of the sentence in order to facilitate syntactic reanalysis computations based on temporal information, they may have made regressions to re-inspect either of these regions. Indeed, adults did make reliably more regressions, *t*1(29) = 2.00, *p* = .05; *t*2(14) = 2.41, *p*<.05, *d* = 0.37, into Region 4 (the verb phrase to which they had incorrectly attached the adverbial phrase) in the early-closure than late-closure condition, although this difference was not reliable in second pass times, *t*1(19) = 2.97, *p*<.01; *t*2(13) = 1.47, *p* = .17. Adults did not, however, make more regressions or exhibit longer reading times into Region 2 (the verb phrase to which the adverbial phrase should have been attached), *t*s ¸1.4; *p*s >.1. This shows that adults inspected the site associated with the syntactic error, not the site associated with the syntactic correction, suggesting that they verified that they had made a mistake, but that they did not reprocess other portions of the preceding sentence in order to restructure the analysis. Note that this finding extends the results reported by Altmann et al. [33], [54], who did not explore eye movement behaviour associated with recovery from the initial garden path. Whilst Altmann et al. study showed longer gaze durations and more frequent regressions out of the target region in the early than late-closure condition, our analyses additionally show where adults target those regressions back to.

To summarise the adult data, there were robust and immediate effects of closure in the predicted direction in the target region. Specifically, adults’ first fixation on the disambiguating adverbial phrase was longer when it was attached to a previously processed clause rather than the currently processed clause. The effects were quite immediate, but also persisted into the later measures of total reading time and second pass time, showing that disruption to processing was substantial. Also in contrast to Experiment 1, adults did not show spillover effects in the post-target region. Finally, adults showed evidence of reanalysis, indexed by more regressions into, and longer re-reading times on the verb to which they had incorrectly attached the adverbial phrase.

### Adults and Children

We then conducted a 3 (group: adults, older children, younger children) ×2 (closure: early-closure, late-closure) mixed design ANOVA. There were reliable effects of group in all measures in all regions (ps <.05) with the following exceptions: there was no effect in the proportion of regressions made out of the target region (*F*s <1.4; *p*s >.2), and no reliable effect in the proportion of regressions made into Region 4, *F*1(2, 55) = 1.59, *p* = .21; *F*2(2, 28) = 8.83, *p*<.01. For all other measures in all regions, adults showed significantly shorter reading times or less frequent regressions than both groups of children (ps <.05). There was one exception to this pattern: in first fixation durations in the post-target region, only the difference between adults and younger children was reliable, *t*1(42) = 2.92, *p*<.01; *t*2(14) = 3.16, *p*<.01. The general pattern of effects is very clear – the main difference in reading behaviour is between adults and children, rather than between children of different ages, and consistent with existing studies, adults are far more efficient in their reading than are children [40], [42], [45]–[47].

There was no effect of closure in first fixation durations (*F*s <1; *p*s >.5) or gaze durations (*F*s <1.3; *p*s >.1). There was no interaction between group and closure in first fixation duration (*F*s <1; *p*s >.5) or in gaze duration, *F*1(2, 55) = 2.67, *p* = .08; *F*2(2, 28) = 1.70, *p* = .20. There was, however, a main effect of closure in the number of first pass regressions made out of the target region, *F*1(1, 55) = 11.87, *p*<.005, η^2^ = .18; *F*2(1, 14) = 11.96, *p*<.005, η^2^ = .46, but no interaction (*F*s <1; *p*s >.5). There was also an effect of closure in go past times, *F*1(1, 55) = 15.14, *p*<.001, η^2^ = .22; *F*2(1, 14) = 8.47, *p*<.05, η^2^ = .38, but no interaction (*F*s <1; *p*s >.8). In second pass times, there was no effect of closure, *F*1(1, 32) = 2.28, *p* = .14; *F*2(1, 12) = .01, *p* = .9, and no interaction, *F*1(2, 32) = .14, *p* = .87; *F*2(2, 12) = 5.91, *p*<.05. Finally, for total reading times, there was a reliable effect of closure with longer reading times in the early-closure condition, *F*1(1, 55) = 16.81, *p*<.001, η^2^ = .23; *F*2(1, 14) = 6.68, *p*<.05, η^2^ = .32, but no interaction (*F*s <1; *p*s >.6).

To summarise the findings in the target region, adults showed very early effects of closure, exhibiting longer first fixations on the adverbial phrase when it was attached to a previous, rather than the current clause. Children also showed early effects, making more leftward eye movements out of the target word to re-read previous portions of the sentence in the early-closure condition. However, as in Experiment 1, they were delayed in the detection of the syntactic ambiguity relative to the adults.

In the post-target region (see Table 4) there was no effect of closure, *F*(1, 55) = 2.51, *p* = .12; F2(1, 14) = .29, p = .6, and no interaction (*F*s <1, *p*s >.5) in first fixation duration. There was, however, a reliable effect of closure in gaze duration, *F*1(1, 55) = 6.33, *p*<.05, η^2^ = .10; *F*2(1, 14) = 9.65, *p*<.01, η^2^ = .41, but no interaction in this measure, *F*2(2, 28) = 2.26, *p* = .12; *F2*<1; *p*>.5.

There was an effect of closure in the number of regressions made out of the post-target region, *F*1(1, 55) = 9.86, *p*<.005, η^2^ = .15; *F*2(1, 14) = 4.75, *p*<.05, η^2^ = .25, but no interaction (*F*s <2, *p*s >.15). In go past times, there was an effect of closure that was significant by participants but not by items, *F*1(1, 55) = 9.06, *p*<.005, η^2^ = .14; *F*2(1, 14) = 2.84, *p* = 2.84, p = .11, η^2^ = .17. However, here there was also an interaction effect which was marginal, *F*1(2, 55) = 2.56, *p* = .087, η^2^ = .09; *F*2(2, 55) = 3.22, *p* = .055, η^2^ = .19. Pairwise comparisons showed that younger children, *t*1(13) = 3.14, *p*<.01; *t*2(14) = 2.37, *p*<.05, *d* = 0.41, but not older children or adults (*t*s <1; *p*s >.3) were exhibiting longer go past times in the post-target region, suggesting effects were delayed, longer-lasting, or both in the youngest age group.

There was also an effect of closure in total reading time in the post-target region, *F*1(1, 55) = 17.13, *p*<.001, η^2^ = .24; *F*2(1, 14) = 9.30, *p*<.01, η^2^ = .40, and a reliable interaction between closure and group, *F*1(2, 55) = 6.43, *p*<.005, η^2^ = .19; *F*2(2, 28) = 3.49, *p*<.05, η^2^ = .20, such that once again younger children showed robust closure effects, *t*1(13) = 3.96, *p*<.005; *t*2(14) = 3.32, *p*<.01, older children showed a non-significant trend towards longer reading times in the early closure condition, *t*1(13) = 1.49, *p* = .16; *t*2(14) = 1.63, *p* = .13, *d* = 0.54, and adults showed no difference (*t*s <1, *p*s >.6). These results indicate that the effect of closure persisted into total reading times in the post-target region for the younger children, to a lesser degree for the older children, but not for the adults, suggesting that the younger children needed additional time to detect and reconstruct their initial misanalysis. In addition, the data suggest a stepwise pattern of effects such that the effect of closure was longer lasting as age decreased. There was no reliable effect of closure and no interaction in second pass reading times in the post-target region (all *F*s <1.6, all *p*s >.2). To summarise, the results from the post-target region reveal that the youngest age group show not only delayed, but also longer-lasting effects of closure than the other two participant groups, suggesting that they need longer to recover from their initial misanalysis. There is also some evidence that older children are intermediate between adults and younger children in that they exhibit longer-lasting disruption effects than adults but shorter-lasting effects than younger children. This presumably reflects how easily each age group recovers from their incorrect analysis, and also perhaps how efficient they are at reanalysis.

Table 5 shows the proportion of regressions made into Regions 2 and 4, and second pass reading times in those same regions. Our analyses for Region 2 showed that there was no effect of closure (*F*s <1; *p*s >.4) in either measure, and there were no reliable interactions (*F*s <1.4; *p*s >.2). Thus, neither children nor adult readers targeted regressive saccades back to Region 2, the main verb of the sentence, when they were garden pathed. The comparable analyses for Region 4, however, did show robust effects. There was a reliable effect of closure, and no interaction (*F*s <2; *p*s >.15), with all groups making more regressions into Region 4 in the early-closure than late-closure condition, *F*1(1, 55) = 9.97, *p*<.005, η^2^ = .15; *F*2(1, 14) = 33.42, *p*<.001, η^2^ = .71, in line with predictions. In second pass times, there was an effect of closure, reliable by participants but not by items, *F*1(1, 40) = 7.47, *p*<.01, η^2^ = .16; *F*2(1, 10) = .71, *p* = .4, η^2^ = .07, but again no interaction (*F*s <1, *p*s >.8). A further, important implication of these data is that when all of the readers experienced disruption to processing they regressed back to the region where they had incorrectly attached the adverbial phrase, not to the site of the correct attachment.

Overall, the results from Experiment 2 show that while all groups of participants showed evidence of syntactic misanalysis in the early-closure condition, the effects increased in immediacy as age increased. Adults exhibited disruption to processing in the early-closure condition on the very first fixation on the target word, while children did not. All groups of participants made more leftward eye movements to re-read previous portions of the text from the target region in the early-closure condition compared with the late closure condition. All participants also spent longer in total reading the adverbial phrase in the early than the late-closure condition. Furthermore, adults, but not children, showed some evidence of reanalysis in the target region, as shown in longer second pass reading times in the early-closure than late-closure condition. However, the youngest children showed very strong effects of closure in the post-target region, showing the effect of closure persisted longer for them compared to the two older age groups. Older children appeared to be intermediary between adults and younger children in their recovery from misanalysis in that the effect for them did not persist to the extent it did for the younger children. Finally, all groups showed increased regressions back to the verb phrase to which they had incorrectly attached the adverbial phrase in the target region. Interestingly, they did not, however, make more regressions into the region which contained the verb phrase to which the adverbial phrase was in fact attached in the early-closure condition (i.e. *I’ll wear* in sentences 2a–2b).

### General Discussion

We conducted two experiments investigating adults’ and children’s processing of syntactic ambiguities during reading. It was predicted that children, like adults, would exhibit disruption while processing garden path sentences in which: (1) a prepositional phrase was attached low to a noun phrase rather high than to a verb phrase; and (2) an adverbial phrase was attached to an earlier verb phrase rather than the immediately preceding clause. It was further predicted that there would be a difference in the time course of these effects: the effects observed would be delayed in children relative to adults, and delayed in younger children relative to older children. Finally, it was predicted that the magnitude of the effects would be greater in younger children than older children, and greater in children than adults. The experiments did not seek to discriminate between different theories of syntactic ambiguity resolution. Rather, they sought to uncover similarities and differences between mature and developing syntactic processing systems during on-line reading.

The data reported from the two experiments support the first of our predictions. All participant groups exhibited effects of attachment and closure in the predicted direction. These results show that adults and children have similar parsing preferences for sentences, and that those preferences are quite strong in children, like adults, in their analysis of certain syntactic structures. On this basis, it might be argued on grounds of parsimony that the cognitive mechanism underlying this preference might be similar in children as in adults. This study is the first to demonstrate garden path effects during normal reading in children, and it is important because it shows a similarity to adults in syntactic performance by the age of seven years in typically developing children.

Our data do not directly address how children of this age develop the same preferences as adults. Indeed, there are several possibilities which are by no means mutually exclusive. On linguistic grounds, the syntactically preferred structures were simpler in that they required fewer nodes in a syntactic tree. Some theories of parsing posit that syntactic complexity determines initial parsing preferences [22], with readers initially pursuing the syntactically simpler analysis. Alternatively, statistical regularities within the language might also explain the effects from the present experiments [55], [56]. Previous research has shown that children pursue the syntactic interpretation that is consistent with verb bias [16], [17], [32]. Thus, whilst it is the case that our sentences in Experiment 1 were syntactically simpler, it is also true that they contained verbs with strong biases towards a VP interpretation.

In relation to the second prediction, adults exhibited more immediate effects of syntactic misanalysis in both experiments than children. While adults showed effects on the target word in both experiments, effects for children first occurred in the post-target region in Experiment 1, and in the target region but in the proportion of regressions made out of the region (compared to in first fixation durations in the adults) in Experiment 2. While children and adults were both garden-pathed, children took a little longer than adults to detect and respond to their initial syntactic misanalysis. To be clear, during the time period within which adults detected their misanalysis, children did not [51], and thus it seems likely that a similar level of syntactic computation takes longer in children than adults.

Let us consider the findings related to our second prediction in more detail. Assuming that adults and children’s planning and execution of saccadic eye movements is influenced by on-going linguistic processing [38], then the data show that the delay between the initial fixation of a word, the detection that that word is syntactically anomalous, and a consequent interruption to on-going saccadic execution is delayed in children relative to adults. In Experiment 1, while adults showed disruption to processing on the target word itself, children did not. Instead, children made a progressive saccade to fixate the post-target region and only then did they show evidence of disruption. That is to say, only later than was the case in the adults was ongoing saccadic behaviour interrupted. In Experiment 2, adults showed evidence of syntactic misanalysis due to an increase in the duration of their very first fixation on the disambiguating word whereas children showed no similar influence during this period. Instead, the first indication of disruption came when they regressed from the critical word. Note that the delay in misanalysis detection in children occurs not only in relation to a specific oculomotor event (i.e., a particular fixation or saccade), but, because children’s fixations are longer on average than those of adults, the period of time between fixation of a critical word and misanalysis detection is also longer. Thus, whilst the relationship between eye movements and linguistic processing in adults is quite tightly synchronous, the relationship in children is less so.

This difference in the time course of effects between adults and children is also relevant to another issue relating to children’s eye movements and reading. Saccadic targeting in children during reading develops quite quickly, with adult-like performance by the age of seven [45], [46], [57]. Even young children target their initial saccades towards the middle of words in an adult-like manner. This behaviour is in contrast to that observed in relation to eye movements associated with linguistic processing difficulty, such as those reported here (and elsewhere [45]). It appears that eye movements relating to basic visual processes during reading (e.g., decisions of where to fixate a parafoveal word) are well developed by the age of 7 years, however, the development of a tightly synchronous relationship between eye movements and linguistic processing such as that observed in adults is not in place by a similar age. Zang et al. [57] have argued that this distinction reflects differences in the rate at which the systems responsible for visual and linguistic processing develop (evidence using magnetoencephalography also supports this hypothesis [58]). They argue that the saccadic targeting system is a low-level reflexive system which develops quickly and reaches maturity early, resulting in adult-like performance in oculomotor targeting strategies (i.e. *where* the eye moves to) by age seven years. In contrast, linguistic processing during reading starts later (when reading instruction begins) and develops much more slowly, and this is evidenced by differences between adults and children in *when* they move their eyes: children need longer than adults to access, identify and incrementally interpret words, resulting in longer reading times and more refixations.

While several computational models have been put forward to describe the mechanisms that underpin eye movements during reading in adults [59]–[64], research that models children’s eye movement behaviour during reading is still in its early stages. Recent findings using reinforcement learning to allow an artificial “agent” to learn to move its eyes to read as efficiently as possible show, consistent with empirical data [45], [57], that adult-like patterns of eye movements (such as fixating close to the word centre and looking longer at longer and more difficult-to-identify words) emerge quite quickly during learning [65], [66].

In addition, more recently Reichle and colleagues [67] have used the E-Z Reader model to simulate various eye-movement phenomena in children (as compared with adults) in order to evaluate different hypotheses about the concurrent development of reading skill and eye-movement behaviour. They found that the principal difference between children and adults was their rate of lexical processing, but that different rates of (post-lexical) language processing also contributed to some phenomena, such as the delayed detection of implausible thematic relations [51]. Importantly, the work by Reichle et al. is consistent with our suggestion that differences between children and adults’ eye movements during reading arise due to a less tight synchrony between linguistic processing and oculomotor control.

Furthermore, our data contribute to the on-going debate in the adult literature regarding whether it is linguistic processing or visual processing that drives eye movements during reading. So-called cognitive models of reading [59]–[62] propose that linguistic processing mediates eye fixations during reading and such models are theoretically consistent with our data. In contrast, oculomotor models [63], [64], [68]–[70] which propose that the eyes are controlled by visuomotor processes, are not consistent with our data. In short, if eye movements in children were primarily driven by visual processing, then why would we observe the differences between adults and children that we do, since saccadic targeting in children is adult-like in proficiency? However, if linguistic processing mediates eye movements during reading, then we should see the types of differences between adults and children that we observe, because development of linguistic processing is delayed in children relative to adults. Our data therefore provide strong support for cognitive models of eye movements during reading.

In relation to the third prediction, we did obtain some evidence suggesting that children exhibited effects of disruption to processing that were of a greater magnitude than adults in both experiments. In Experiment 1, children showed numerically more regressions out of the post-target region in the low-attached than high-attached condition compared to adults. In Experiment 2, children showed numerically longer gaze durations and go past times in, and more regressions out of, the post-target region for the early than the late closure sentences than did adults. In addition, Experiment 2 revealed more enduring disruption to processing for younger children following their initial syntactic misanalysis: younger children showed a reliable effect of closure in total reading times in the post-target region while older children and adults did not (although older children showed a numerical trend). This shows that it not only takes longer for children to detect an initial syntactic misanalysis, but also that once their misparse has been detected, younger children take longer than adults to recover from it.

Related to this point, there were some suggestions in Experiment 1 of more efficient reanalysis in the adult readers than the children: adults spent longer re-reading the noun phrase after encountering (but before going past) the disambiguating region in the low- than high-attached condition. That is, on encountering the prepositional phrase (*with the trunk* in Sentence 1b) in the low-attached condition, adults regressed back and re-read the correct attachment site (*the elephant* in Sentence 1b) more than in the high-attached condition. Such behaviour is likely to reflect syntactic reanalysis. In contrast to the adults, children showed no evidence of more regressions back to, or longer go past times on either the verb phrase or the noun phrase in Experiment 1. Together, differences in magnitude, persistence and patterns of regressions between adults and children suggest that children fail to recover from initial misanalyses as effectively or as efficiently as adults. This suggestion is in accord with findings from Trueswell et al. [15], [71], whose visual world studies showed that children were less effective than adults at revising initial parsing commitments, and tended to persist with their original (syntactically simpler) analysis. We also know that children have poorer cognitive control than adults [72] due to the on-going development of executive function abilities throughout childhood [73]. Executive functions refer to a set of cognitive processes that underlie goal-directed behaviour and encompass working memory, cognitive flexibility, planning and inhibitory control. One or more aspects of executive function may account for children taking longer to reject and recover from their initial misanalysis. Novick and colleagues [74] proposed that executive function, in particular inhibitory control, plays an important role in language processing when the initial interpretation of a sentence has to be inhibited and replaced by an alternative interpretation. Recent evidence from pre-school children indeed suggests that performance on a Go-No Go task, tapping inhibitory control (but not performance on tasks which tapped cognitive flexibility, working memory or planning), is associated with garden path effects in an act-out version of the Trueswell kindergarten path studies [75]. It may be, then, that the children in our study were less able to inhibit their initial interpretation, making the construction of an alternative analysis more difficult for them.

Poor inhibitory control occurs alongside reduced working memory capacity in children [76], and this may also contribute to children’s less efficient recovery from initial syntactic misanalysis. We know that a critical role of working memory in reading is to update relevant information, while suppressing non-relevant information [77], and while children’s poorer inhibitory control may lead them to perseverate with their initial incorrect analysis, their reduced working memory capacity may also mean that they have fewer resources available to construct an alternative.

Finally, it may also be the case that adults have better spatial awareness of the text, or have increased resources to dedicate to accurately directing their saccades back to informative regions to aid reanalysis, than is the case for the children. This might especially be the case when informative regions occur early in the sentence and are not immediately proximal to the region of the sentence at which the initial misanalysis is detected. This was the case in Experiment 1, for which we saw the most pronounced differences between adults and children. Note that for any individual regressive eye movement, the most likely landing site is on the word immediately to the left of the launch site [78]. Thus, in the present experiment, it is more likely that the syntactic structures used in Experiment 1 would show differences between children and adults in relation to regressive saccadic targeting than those used in Experiment 2. Consistent with this argument, (although we know that adults do not always make linguistically-guided regressions [79]–[81]) it may be the case that while adults make comparatively accurate regressions back to the source of error to perform “linguistically informed” reanalysis of the sentence [2], [82] children’s regressions may be better described as a delaying tactic used to provide “time out”, rather than time spent in directed linguistic reanalysis [78].

In summary, the two experiments reported in this paper showed that children misanalysed sentences for which we know there is a strong processing bias in adults. Furthermore, while adults showed more immediate effects of initial misanalysis of syntactic ambiguity than children, children (and especially younger children) tended to exhibit longer-lasting effects of a greater magnitude. Finally, there is some evidence that children are less efficient in their reanalyses of an initial incorrect interpretation than adult readers. The results strongly suggest that children and adults have in place a similar underlying processing mechanism for syntactic analysis but that this operates with a slower time course. Children also appear to be less flexible than adults in their syntactic reanalyses, and we have suggested that this could be due to their weaker inhibitory control and/or reduced working memory capacity. More generally, the data are consistent with the suggestion that eye movement behaviour associated with decisions as to where to initially target saccades is more adult-like than eye movement behaviour reflecting on-going linguistic processing. This is likely due to differences in the rate of development of the visual processing system compared with the linguistic processing system [57].
